# Cloning and Characterization of *TaPP2AbB"-α*, a Member of the PP2A Regulatory Subunit in Wheat

**DOI:** 10.1371/journal.pone.0094430

**Published:** 2014-04-07

**Authors:** Dan Liu, Ang Li, Xinguo Mao, Ruilian Jing

**Affiliations:** National Key Facility for Crop Gene Resources and Genetic Improvement/Institute of Crop Sciences, Chinese Academy of Agricultural Sciences, Beijing, China; China Agricultural University, China

## Abstract

Protein phosphatase 2A (PP2A), a major Serine/Threonine protein phosphatase, consists of three subunits; a highly conserved structural subunit A, a catalytic subunit C, and a highly variable regulatory subunit B which determines the substrate specificity. Although the functional mechanism of PP2A in signaling transduction in *Arabidopsis* is known, their physiological roles in wheat remain to be characterized. In this study, we identified a novel regulatory subunit B, TaPP2AbB"-α, in wheat (*Triticum aestivum* L.). Subcellular localization indicated that TaPP2AbB"-α is located in the cell membrane, cytoplasm and nucleus. It interacts with both TaPP2Aa and TaPP2Ac. Expression pattern analyses revealed that *TaPP2AbB"-α* is strongly expressed in roots, and responds to NaCl, polyethylene glycol (PEG), cold and abscisic acid (ABA) stresses at the transcription level. Transgenic *Arabidopsis* plants overexpressing *TaPP2AbB"-α* developed more lateral roots, especially when treated with mannitol or NaCl. These results suggest that *TaPP2AbB"-α*, in conjunction with the other two PP2A subunits, is involved in multi-stress response, and positively regulates lateral root development under osmotic stress.

## Introduction

Environmental stresses, such as water deficit and high salinity, are major challenges for plant growth and development. Plants adapt to severe environments by regulating morphogenesis and physiological reactions. Increased lateral root formation is one strategy for plants to survive in unfavorable conditions [1], [2]. More lateral roots permit plants to absorb nutrients and water more easily in order to overcome stress damage, especially under osmotic stress [3]–[5]. Many genes are involved in plant stress response [6]; among them, protein kinases and protein phosphatases are basic components of stress signal transduction [7]. For example, ABA-activated SNF1-related protein kinases (SnRK) phosphorylate downstream substrates to enhance drought tolerance in *Arabidopsis*, whereas, SnRKs are inhibited by protein phosphatases in the absence of ABA [8]–[10]. Protein kinases have been well described in wheat, but the roles of protein phosphatases (PPs) have not been investigated as widely [11], [12].

PP2A is one of the most important Serine/Threonine (Ser/Thr) protein phosphatases that regulate many cellular processes, such as transcription, translation, the cell cycle, metabolism, and apoptosis [13], [14]. PP2A positively regulates salt stress response through modulating polar auxin transport (PAT). It dephosphorylates auxin efflux carrier PIN proteins, which can be regulated by Ser/Thr kinase PINOID, and influences the distribution of auxin [15]–[17].

As a heterotrimeric phosphase, PP2A consists of a scaffolding subunit A (PP2Aa), a regulatory subunit B (PP2Ab), and a catalytic subunit C (PP2Ac). In *Arabidopsis,* PP2Aa has three isoforms, PP2Aa1, PP2Aa2 and PP2Aa3, which are composed of tandem HEAT repeats that form a hook-like structure for binding PP2Ac and PP2Ab [18]. Auxin transportation is affected in the mutant *rcn1* (root curl in naphthylphthalamic acid1), which exhibits a root coiling phenotype and belongs to the *PP2Aa* group [19]. Two single mutants (*pp2aa2* and *pp2aa3*) and one double mutant (*pp2aa2 pp2aa3*) in *Arabidopsis* develop normally, but plants exhibit serious developmental aberrations when *PP2Aa1* is absent [15]. PP2Ac has five isoforms grouped into two subfamilies based on their conserved sequences. Subfamily I (PP2Ac1, PP2Ac2, and PP2Ac5) is involved in stress response, and the ABA and brassinosteroid (BR) signaling pathways. Subfamily II (PP2Ac3 and PP2Ac4) participates in dephosphorylation of PIN proteins in the auxin signaling pathway [20]. Methylated PP2Ac dephosphorylates receptor BRI1 to attenuate BR signaling [21]. Regulatory subunit B of PP2A (PP2Ab) determines multiple roles of PP2A in different signaling pathways.

Most researches on PP2Abs have been performed on animals where four gene families encode PP2Abs, including B (B55, PR55, PPP2R2), B′ (B56, PR61, PPP2R5), B″ (PR72, PPP2R3), and B′″(PR93/PR110) [22], [23]. Down-regulation of PR55/Bδ resulted in a diminished eye and enlarged trunk in gold fish [24]. Knockdown of PR61/B′δ in mice led to decreased glycogen synthase kinase 3β (GSK3β) activity, increased cyclin-dependent kinase 5 (CDK5) activity in the brain, and reduced ability to perform beam walking [25]. PP2AbB″ containing two calcium binding sites formed by EF-hands is involved in the adenosine 5′-monophosphate (AMP)-activated protein kinase (AMPK) signaling pathway through which it regulates the activity of PP2A in pig [26]. In *Arabidopsis*, at least 17 PP2Ab have been reported, including two PP2AbB, nine PP2AbB′, five PP2AbB″ and TAP46 [27]. Only a few PP2Ab are well studied. The expression of *TAP46* is induced by low temperature suggesting a role in cold stress signaling in *Arabidopsis* [28]. TON2, a member of the *Arabidopsis* PP2AbB″ subfamily, has a role in determining the geometry of microtubule nucleation and organization of the cortex [29]. Some members of B' regulatory subunits of PP2A dephosphorylate BZR1 thus promoting plant growth [30]. Although PP2Abs determine substrate specificity and subcellular location of PP2A [31], detailed studies in *Arabidopsis* are restricted to TAP46 and TON2. PP2Ab in wheat has not been well characterized. *TaB-β* (PP2AbB) was isolated and shown to be involved in a stress signaling pathway [32]. Some sequence information on the regulatory B″-α subunit was characterized in *Oryza sativa*, *Setaria italica* and *Brachypodium distachyon*, but the functions of these genes are largely unknown.

The present research focused on the function of a novel PP2A regulatory subunit B″ gene *TaPP2AbB"-α* in wheat. We isolated *TaPP2AbB"-α* based on information from the wheat D genome sequence and the candidate sequence from *Oryza sativa*. We found that TaPP2AbB"-α interacted with PP2Aa and PP2Ac, which were isolated from wheat. Expression of *TaPP2AbB"-α* was up-regulated by multiple stresses. *Arabidopsis* lines overexpressing *TaPP2AbB"-α* developed more lateral roots and grew better than the wild type under the medium osmotic stress, presenting a possibility of using *TaPP2AbB"-α* in transgenic breeding to improve abiotic stress tolerance in crop plants.

## Materials and Methods

### Plant materials, growth conditions and stress treatments

Drought-tolerant common wheat (*Triticum aestivum* L.) cultivar Hanxuan 10 was used as the plant material. Ten-day-old seedlings (two-leaf stage) grown in 1/2 MS liquid culture at 23°C in a 12 h light/12 h darkness photoperiod were subjected to the following four treatments: 16.1% PEG (−0.5 MPa), 250 mM NaCl, 4°C, and 50 µM ABA. The leaf (seedlings sprayed with ABA) and root samples (treated with NaCl, PEG and 4°C) were harvested at 0, 0.5, 1, 1.5, 2, 3, 6, 12, 24, 48 and 72 h after each treatment, and tissues were stored at −70°C for RNA isolation and transcription level detection. Samples harvested at 0 h were used as controls for the corresponding treatments. Total RNAs was extracted using Trizol reagent.

### Cloning and sequencing of full-length *TaPP2AbB"-α* cDNA

The putative full-length cDNA of *TaPP2AbB"-α* was obtained using the candidate sequence from *Oryza sativa* (NM_001071385.1) and sequence information for a dehydration-inducible cDNA library of wheat D genome-bearing *Aegilops tauschii* [33]–[35]. The full-length cDNA of *TaPP2AbB"-α* was amplified using primers: *TaPP2AbB"-α* F: 5’ TTGACGGCATGGAGGTG 3’, *TaPP2AbB"-α* R: 5’ GTAGCACTCACATGATTCAGAAT 3’. The cDNA of Hanxuan 10 as templates, and PCR products were ligated with pEASY-blunt simple vectors (TransGen; Beijing, China). The ligated products were transformed into *E-coli* DH5α, and then positive recombinants were sequenced with an ABI 3730XL 96-capillary DNA analyzer (Lifetech; USA).

A Neighbor–Joining tree was constructed by MEGA5.05 software [36]. AtPP2AbB"-β (NP_851089.1), GmPP2AbB"-α like (XP_003556377.1), VvPP2AbB"-α (XP_002272394.1), SiPP2AbB"-α like (XP_004982873.1), OsPP2AbB"-α (NP_001064850.1), MtPP2AbB"-γ (XP_003609619.1), BdPP2AbB"-γ like (XP_003568929.1), GmPP2AbB"-γ like (XP_003529585.1), *Xenopus laevis* PR74 (AAK98641), *X. laevis* PR130 (NP_001082623.1), *Mus musculus* PP2AbB" isoform 1 (NP_001154834.1), *Homo sapiens* PP2AbB" isoform 1 (NP_002709.2), *Macaca mulatta* PP2AbB"-α (NP_001244537.1) and TaPP2AbB"-α were used to construct the tree.

Database (nucleotide and protein blasts) searches were performed through the NCBI website. Sequence alignments and comparisons were implemented by the MegAlign program in DNAStar and DNAman. Protein predictions were performed using Softberry (http://www.softberry.com).

### Subcellular localization of TaPP2AbB"-α protein

The ORF of *TaPP2AbB"-α* without the termination codon was amplified using forward primer: 5’ CCC***AAGCTT***ATGGAGGTGGAAGCGGC 3’ (*Hin*d III site in bold italics), and reverse primer: 5’ ACGC***GTCGAC***GAATGGAGCTTCAAGTGACTCG 3’ (*Sal* I site in bold italics), then inserted into the pJIT163-GFP vector using T4 ligase.

The 10-day-old wheat seedling was used for protoplast isolation. The leaf strips of 0.5–1 mm length were cut from the middle part of leaves, and digested in cellulose R10 and macerozyme R10 enzyme solution buffer for 3 h to obtain wheat mesophyll protoplast cell. The fusion construct (TaPP2AbB"-α-GFP) and control (GFP) were transformed into wheat mesophyll protoplast cells by the PEG-mediated method [37]. After incubation in W5 nutrient solution at 23°C for 16 h, the protoplast cells were examined using a laser scanning confocal microscope (Leica TCS-NT; Germany). GFP auto-fluorescence was collected in the range of 500–570 nm wavelength. For chloroplast auto-fluorescence, the wavelength range measured was 630–700 nm. Under which wavelength, the chloroplasts were shown in red color.

### Interactions of TaPP2AbB"-α with TaPP2Aa and TaPP2Ac

Yeast two-hybrid and firefly luciferase complementation imaging assays were carried out to identify physical interaction of TaPP2AbB"-α with both TaPP2Aa and TaPP2Ac.

The *TaPP2AbB"-α* coding region was amplified using primer pair: Forward: 5’ CA***CCATGGC***CGATGGATGGAGGTGGAAGCGGC 3’ (*Nco* I site in bold italics) Reverse: 5’ ACGC***GTCGAC***TCAGAATGGAGCTTCAAGTGAC 3’ (*Sal* I site in bold italics) for the pGBKT7 vector construct. Full length cDNAs of *TaPP2Aa* and *TaPP2Ac* were separately sub-cloned into the pGADT7 vector. Two pairs of vectors, *TaPP2AbB"-α*-pGBKT7/*TaPP2Aa*-pGADT7, and *TaPP2AbB"-α*-pGBKT7/*TaPP2Ac*-pGADT7 were transformed into yeast strain AH109. Transformations were selected on SD/–Trp/–Leu screening medium with X-β-gal (5-bromo-4-chloro-3-indolyl-β-D-galactopyranoside). The interaction was tested on SD/–Trp/–Leu/–His plates containing 5 mM 3-AT (3-amino-1,2,4-triazole). Yeast cells on SD/–Trp/–Leu plates were used as controls for co-transformation.

For the firefly luciferase complementation imaging assay, which is an effective method to detect protein-protein interaction [38], full length cDNAs of *TaPP2AbB"-α* and *TaPP2Aa* were separately PCR-amplified and ligated into the pCAMBIA-cLuc and pCAMBIA-nLuc vectors. The constructed vectors were transformed into *Agrobacterium tumefaciens* strain GV3101, and further transformed into *Nicotiana benthamiana* leaves. After 3 days, the leaves were sprayed with 1 mM luciferin, and kept in darkness for 10 min. A camera fitted with a low-light cooled charge-coupled device was employed to capture the LUC (luciferase) image (Nikon-L936, Andor Tech; UK). The exposure time for LUC images was 15 min. ABA-insensitive 1 (ABI1) and its substrate Open Stomata 1 (OST1), from *Arabidopsis* were used as positive controls [39].

### Expression pattern of *TaPP2AbB"-α* in wheat

Quantitative real-time PCR (qRT-PCR) was performed to determine the expression pattern of *TaPP2AbB"-α*. Actin transcript was used as an internal control to quantify the relative transcript level. The qRT-PCR was performed in triplicate with an ABI PRISMH 7900 system (Applied Biosystems, USA) using the SYBR Green PCR master mix kit (Takara, Japan). The specific primers (F: 5’ CTGGCTCTCCCCGTGTTATG 3’, R: 5’ AGAGGAGATCCCAAGGATGATG 3’) were designed according to the full-length of the *TaPP2AbB"-α* cDNA sequence. The relative level of gene expression was detected using the 2^−△△*C*T^ method [40]. △△*C*_T_  =  (*C*_T, Target_ - *C*_T, Actin_)_Time x_ - (*C*_T, Target_ - *C*_T, Actin_)_Time__0_. The *C*_T_ (cycle threshold) values for both the target and internal control genes were the means of triplicate independent qRT-PCRs. Time x represented the treatment time point (0.5, 1, 1.5, 2, 3, 6, 12, 24, 48 and 72 h), and time 0 h represented the time immediately prior go treatment. Expression of *TaPP2AbB"-α* in seedling leaves was regarded as standard due to its lowest expression level in that tissue. The corresponding formula was modified to △△*C*_T_  =  (*C*_T, Target_ - *C*_T, Actin_)_leaf_ - (*C*_T, Target_ - *C*_T, Actin_)_root_, to identify the expression pattern of *TaPP2AbB"-α* in leaf and root tissues from wheat seedlings.

### Generation of transgenic plants in *Arabidopsis*

*TaPP2AbB"-α* cDNA containing the entire ORF was inserted into pCHF3 under control of the CaMV 35S promoter and NOS terminator, using primers 5’ GG***GGTACC***ATGGAGGTGGAAGCGGC 3’ (*Kpn* I site in bold italics) and 5’ CG***GGATCC***TCAGAATGGAGCTTCAAGTGAC 3’ (*Bam*H I site in bold italics). The p35S-*TaPP2AbB"-α*-*NOS* construct and the p35S-NOS vector were separately transformed into *Agrobacterium* strain GV3101 and then infected into wild type *Arabidopsis* plants by floral infiltration. Positive T3 generation transgenic *Arabidopsis* plants overexpressing *TaPP2AbB"-α* were screened by kanamycin plates and PCR amplification.

### Morphological characterization of transgenic *Arabidopsis*

*Arabidopsis* seeds were sown on MS medium solidified with 0.8% agar. Seeds were vernalized for 36 h at 4°C before being transferred to a growth chamber. To examine root morphology, the 8-day-old seedlings vertically grown on normal MS solid medium were replanted to MS medium plates containing NaCl (50, 100 and 150 mM), and mannitol (100, 200 and 300 mM). Root system morphologies were examined 3 days after replanting. The root length of *Arabidopsis* was measured with a ruler. The number of lateral root was captured with EXPSON EXTRESSION 10000XL (EXPSON, Japan) and counted with winRHIZO software.

## Results

### Isolation and sequence analysis of *TaPP2AbB"-α*

**Figure 1:**
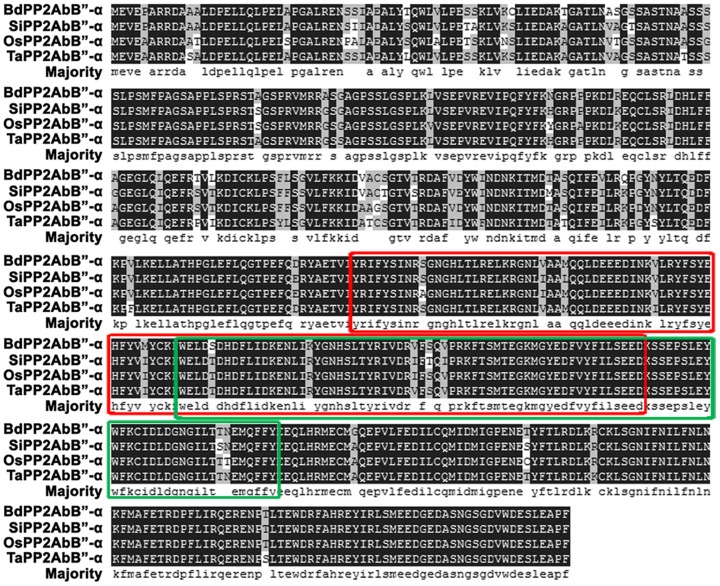
Alignment of the predicted amino acid sequences of PP2AbB"-α from different plant species. Alignment was performed according to DNAman. Common identical amino acid residues are shown in black background. The FRQ1 domain is marked in red rectangles, and the EF-hand-5 domain is marked in green rectangles. Abbreviations on the left side of each sequence: Bd, *Brachypodium distachyon*; Si, *Setaria italica*; Os, *Oryza sativa*; Ta, *Triticum aestivum*.

*TaPP2AbB"-α* is 1,648 bp in length, consisting of an 8 bp 5’ untranslated region, a 1,626 bp ORF, and a 14 bp 3’ untranslated region. The ORF encodes 541 amino acid residues (AAR) with a calculated molecular mass of 62 kDa and predicted pI of 4.92. TaPP2AbB"-α has two domain structures, characterized by the EF-Hand-5 (330–422 AAR) and FRQ1 (272-391 AAR) domains that belong to the EF-hand superfamily. The deduced amino acid sequence shows high homology with counterpart PP2AbB"-α family members from other plant species, viz. *Oryza sativa* (NP_001064850.1), *Setaria italica* (XP_004982873.1) and *Brachypodium distachyon* (XP_003574057.1) (Figure 1). We also performed protein blast in the website of TAIR (http://www.arabidopsis.org/Blast/index.jsp). Several amino acid sequences were identified, sharing about 70% similarity with TaPP2AbB"-α. But, the classification of these amino acid sequences from *Arabidopsis* was unclear.

### Phylogenetic analysis

**Figure 2:**
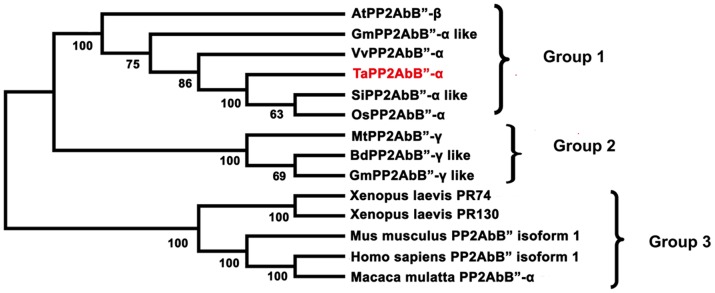
Phylogenetic tree of TaPP2AbB" from wheat and PP2Ab from other species. This phylogenetic tree performed by MEGA 5.05 is based on putative amino acid sequences. Bootstrap values are in percentages. There are three distinct isoform groups in the phylogenetic tree. At, *Arabidopsis thaliana*; Gm, *Glycine max*; Vv, *Vitis vinifera*; Ta, *Triticum aestivum*; Si, *Setaria italica*; Os, *Oryza sativa*; Mt, *Medicago truncatula*; Bd, *Brachypodium distachyon*.

A phylogenetic tree was constructed with the putative amino acid sequences of TaPP2AbB"-α and members of the PP2AbB" family in other species. PP2AbB"-α from animals and plants were in two distinct clades (Figure 2). TaPP2AbB"-α and its counterparts from plants were placed into Group 1; PP2AbB"-γ in Group 2 were in one clade, and PP2AbB"-α from animals were in another (Group 3).

### Subcelluar localization of TaPP2AbB"-α protein

**Figure 3:**
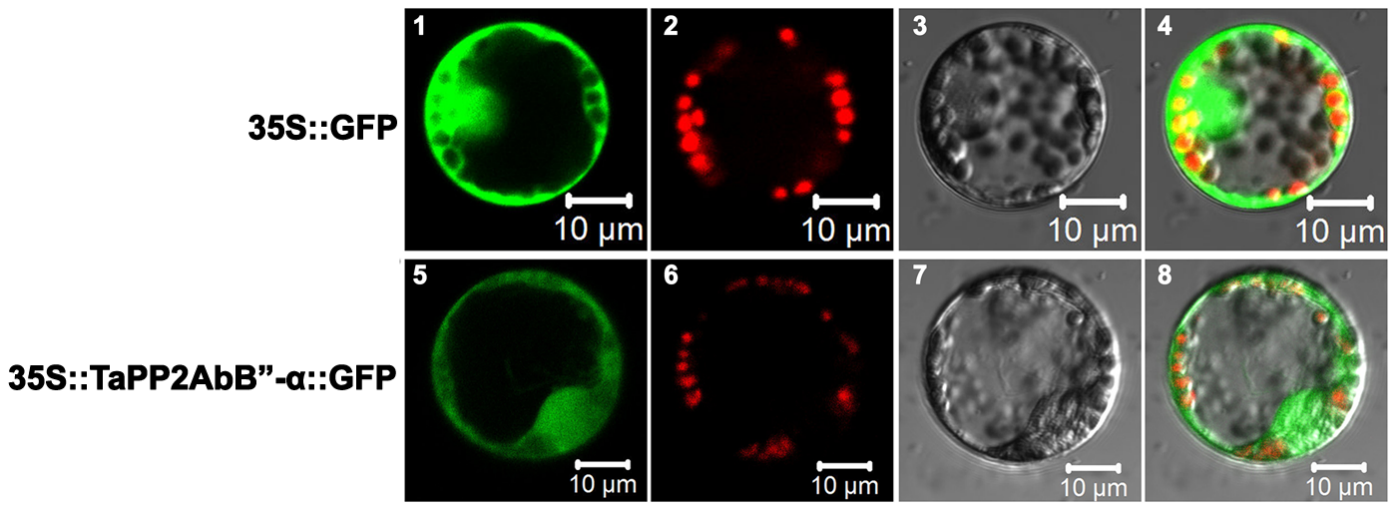
Subcellular localization of PP2AB”-α in wheat protoplast cells. GFP and TaPP2AbB"-α-GFP fusion proteins were transiently expressed under control of the CaMV 35S promoter in wheat protoplast cells and observed with a laser scanning confocal microscope. Images are taken in dark field for green fluorescence (1, 5). The chloroplasts (2, 6) are indicated by red autofluorescence. The cell outline (3, 7) and the combination (4, 8) are photographed in bright field. Scale bar  =  10 µm.

Wheat protoplast cells were used to check subcellular localization of TaPP2AbB"-α. The deduced amino acid sequence contains no putative nuclear localization sequence (NLS) site or transmembrane region. The fusion construct *TaPP2AbB"-α::GFP* driven by the CaMV 35S promoter was transiently expressed in living wheat protoplast cells. As predicted, TaPP2AbB"-α-GFP was present in the cell membrane, cytoplasm and nucleus (Figure 3).

### Phenotypes of overexpression lines of *TaPP2AbB"-α* in *Arabidopsis*

**Figure 4:**
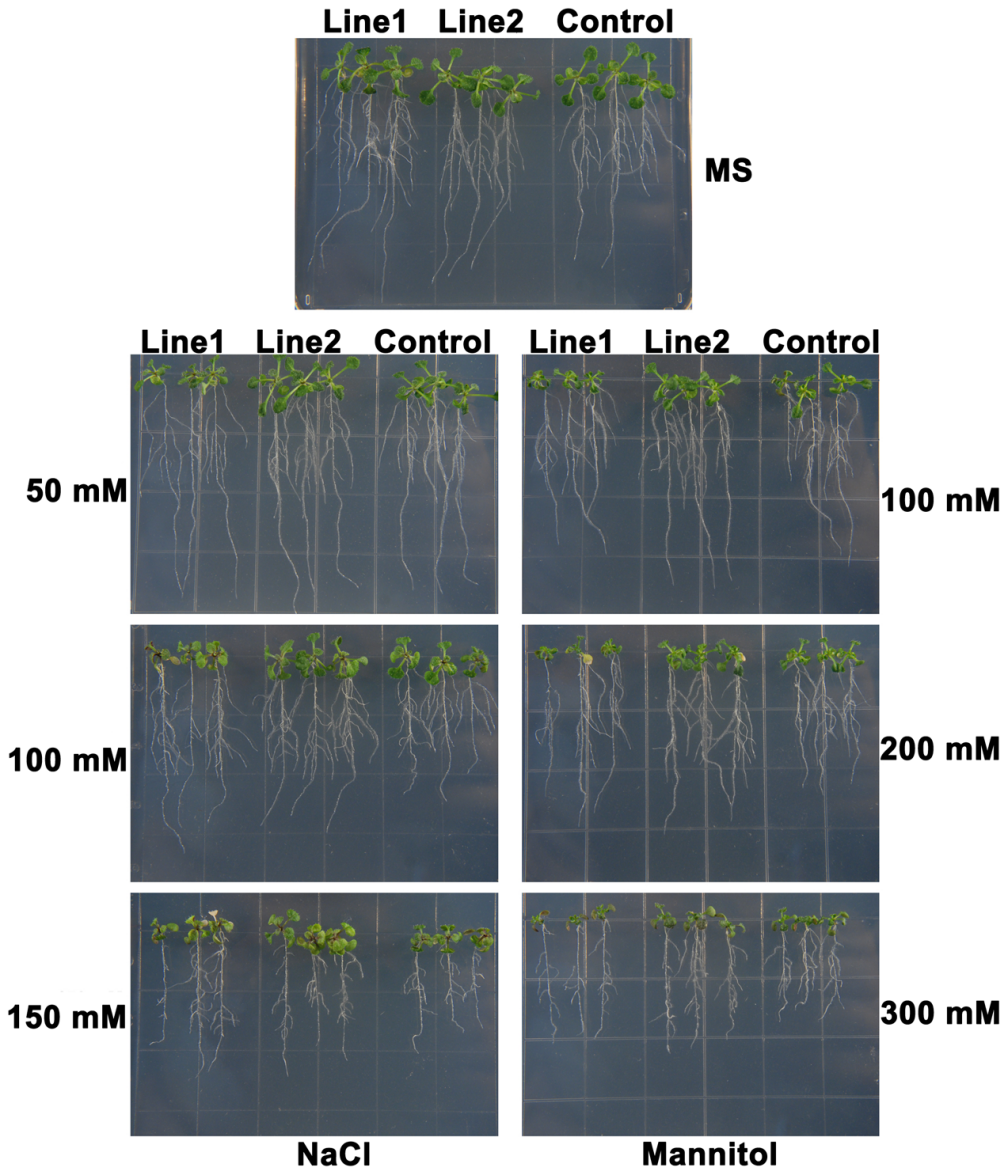
Overexpression of *TaPP2AbB"-α* promotes root growth in *Arabidopsis*. Root morphologies of *TaPP2AbB"-α* overexpression lines and control (p35S-NOS vector plants) grown on MS medium, and MS medium with added NaCl and mannitol for 3 days after replanting.

**Figure 5:**
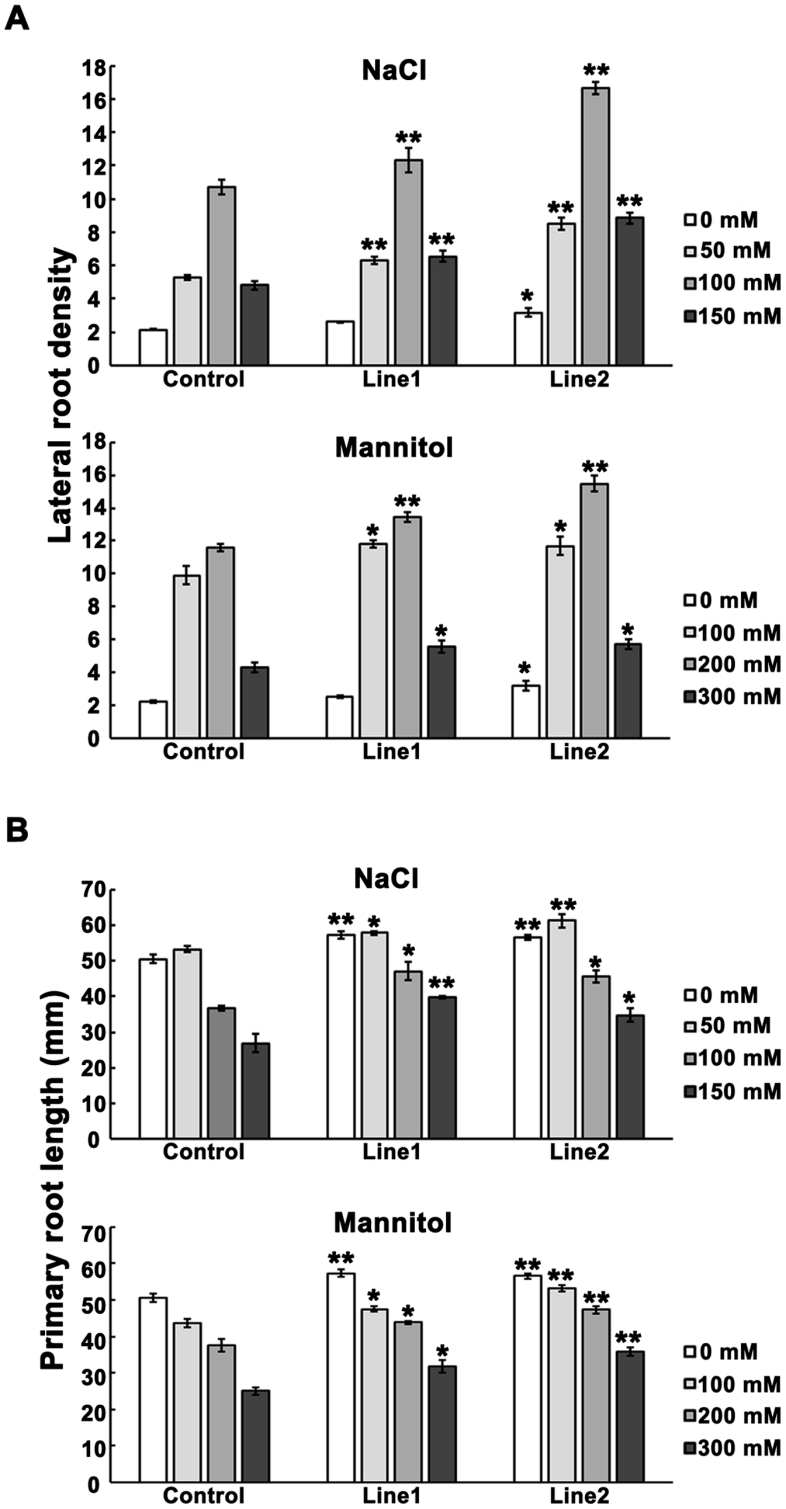
Overexpression of *TaPP2AbB"-α* promotes root growth in *Arabidopsis* as exhibited by more lateral roots and longer primary roots. (A) Lateral root density (number of lateral roots/cm primary root) of *TaPP2AbB"-α* overexpression lines and control. (B) Primary root length of *TaPP2AbB"-α* overexpression lines and control. *, significantly different at *P*  =  0.05; **, significantly different at *P*  =  0.01. Data represent mean ± SE (n = 9).

To evaluate the possible function of *TaPP2AbB"-α*, transgenic plants were assessed under different abiotic stress conditions. Morphological assays indicated differences in root systems between transgenic and control plants (the p35S-NOS vector plants) (Figure 4). Overexpression lines grew better, and developed more lateral roots, especially under the stresses of mannitol and NaCl (Figure 5A). Primary roots of overexpression lines were significantly longer than those of control plants under osmotic stress (Figure 5B). These results suggested that *TaPP2AbB"-α* may be involved in stress response and regulation of root development, especially lateral root densities under 100 mM NaCl and 200 mM mannitol treatments.

### Expression pattern of *TaPP2AbB"-α* in wheat

**Figure 6:**
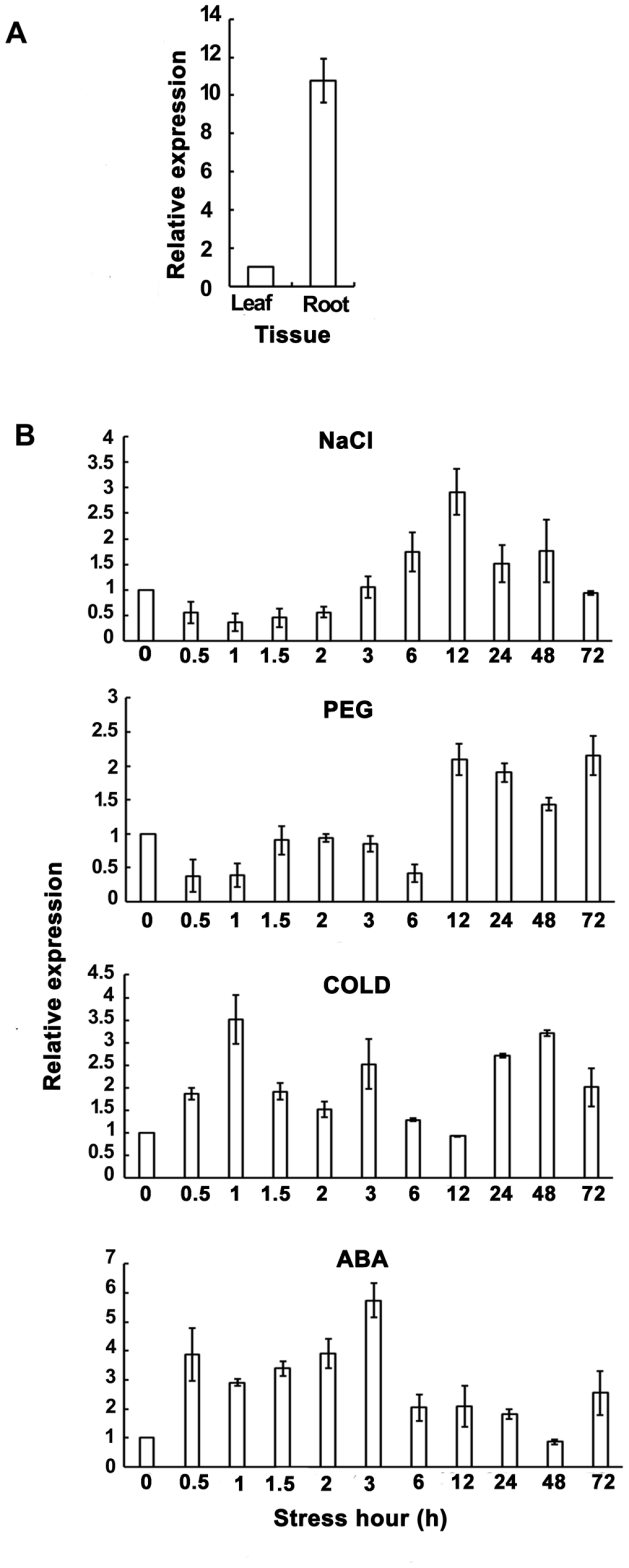
Expression patterns of *TaPP2AbB"-α* in different wheat seedling tissues after subjection to various stresses. (A) Expression pattern of *TaPP2AbB"-α* in roots and leaves of wheat seedlings. (B) Expression patterns of *TaPP2AbB"-α* under various stress conditions. Actin was used as an internal control. Vertical columns indicate relative transcript levels. Data represent mean ± SE of three replicates.

*TaPP2AbB"-α* was expressed strongly in roots, and weakly in leaves of wheat seedlings (Figure 6A). Various up-regulated expression patterns occurred under diverse abiotic stresses (Figure 6B). Under NaCl stress, the expression of *TaPP2AbB"-α* decreased during the first 2 hours, the transcription level increased and peaked at 12 h, and then decreased. Under PEG stress, the transcription level decreased during the first hour, increased from 12 h, slightly decreased at 48 h, and increased again at 72 h. Under cold stress, expression increased rapidly in the first hour, and the level peaked at 3 h, 24 h and 48 h. With ABA treatment, the expression level of *TaPP2AbB"-α* increased rapidly and reached a maximum at 3 h, then quickly decreased to the 0 h level.

### TaPP2AbB"-α interacts with both TaPP2Aa and TaPP2Ac

**Figure 7:**
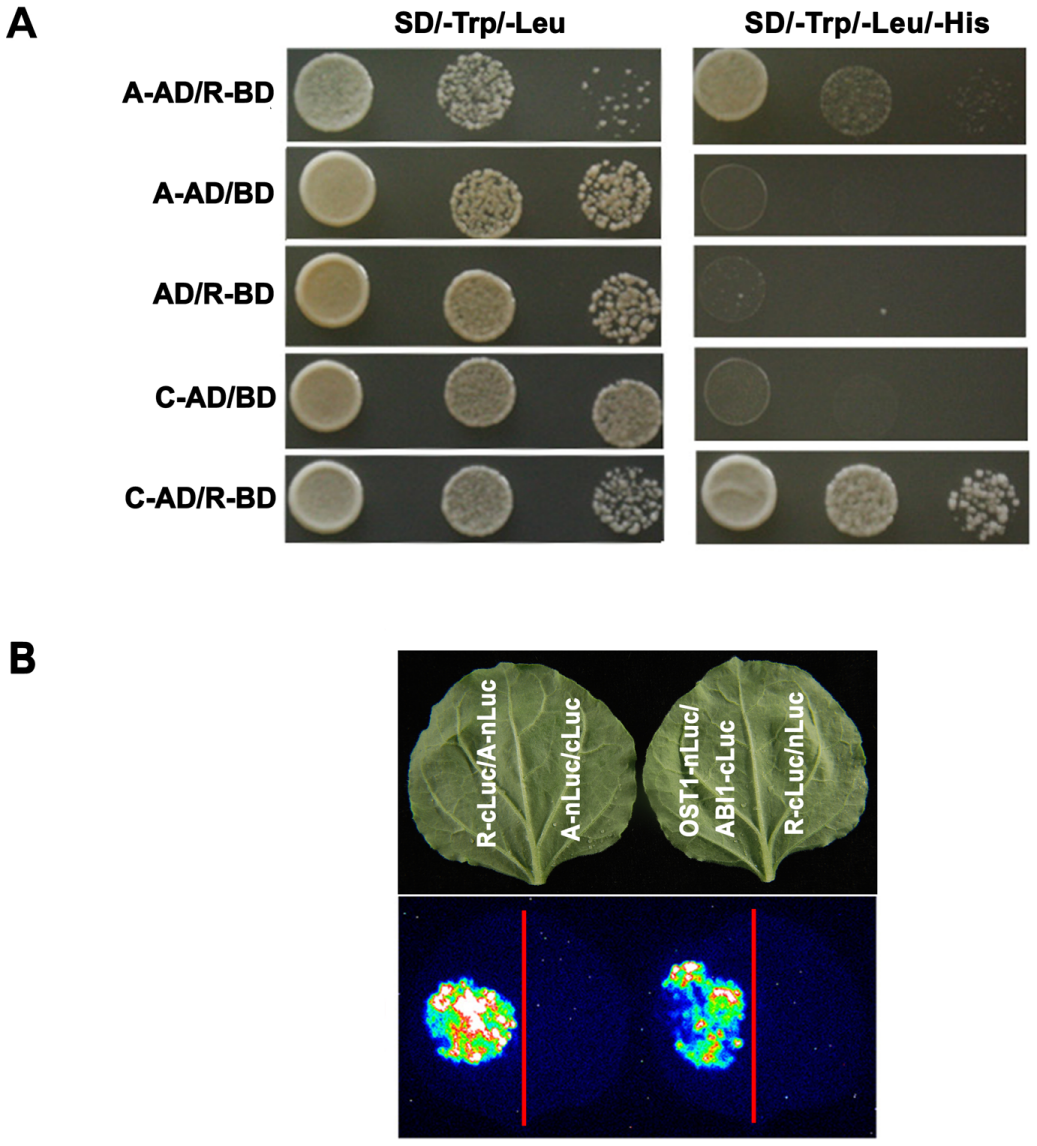
TaPP2AbB"-α interacts with TaPP2Aa and TaPP2Ac. (A) Yeast two-hybrid assay shows interactions between TaPP2AbB"-α and subunits A and C of PP2A. Abbreviations on the left side are: AD, pGADT7 vector; BD, pGBKT7 vector; R, TaPP2AbB"-α; A, TaPP2Aa; C, TaPP2Ac. (B) Interaction of TaPP2AbB"-α with TaPP2Aa revealed by firefly luciferase complementation imaging assay in *Nicotiana benthamiana* leaves. A, TaPP2Aa; R, TaPP2AbB"-α. Different fluorescence intensity resulted in the color-mixed spots.

Yeast cells co-transformed with the *TaPP2AbB"-α*-pGBKT7/*TaPP2Aa*-pGADT7, *TaPP2AbB"-α*-pGBKT7/*TaPP2Ac*-pGADT7, and *TaPP2AbB"-α*-pGBKT7/pGADT7 vector, and with *TaPP2Aa*-pGADT7/pGBKT7 and *TaPP2Ac*-pGADT7/pGBKT7 grew well on SD/–Trp/–Leu plates. Yeast cells co-transformed with the *TaPP2AbB"-α*-pGBKT7/pGADT7 vector, *TaPP2Aa*-pGADT7/pGBKT7 and *TaPP2Ac*-pGADT7/pGBKT7, could not grow on SD/–Trp/–Leu/–His with 5 mM 3-AT (Figure 7A). These results indicated that TaPP2AbB"-α interacted with both TaPP2Aa and TaPP2Ac. In the firefly luciferase complementation imaging assay, the image of LUC was captured during co-transformation of *TaPP2AbB"-α*-cLuc/*TaPP2Aa*-nLuc as well as the positive control in *Nicotiana benthamiana* leaves (Figure 7B). Different fluorescence intensity resulted in the color-mixed spots. These results confirmed that TaPP2AbB"-α interacts with TaPP2Aa.

## Discussion

The PP2A regulatory subunit is responsible for substrate specificity and localization of PP2A [41]. Approximately fifteen and seventeen B subunits have been identified in vertebrates and plants, respectively. All B subunit members are derived from four diverse gene families, and little sequence similarity exists between families while maintaining high sequence similarity within families [22]. In this study, the identified wheat PP2Ab, designated as TaPP2AbB"-α, has the same EF-hand domain as PP2AbB" from animals and plants.

We used the cDNA sequence of *TaPP2AbB"-α* to blast in wheat A genome and D genome. There are 12 regions in *TaPP2AbB"-α* matching with scaffold59526 of A genome and scaffold72594 of D genome, respectively. The 12 regions of *TaPP2AbB"-α* almost covered the whole coding region. Most part of the 12 regions have 97% identity with scaffold59526 and scaffold72594. We presumed that TaPP2AbB"-α was exist in A genome and D genome. What’s more, we did not find other scaffolds similar to TaPP2AbB"-α. This phenomenon may imply that the TaPP2AbB"-α has no homologue in wheat.

Our data indicates that transgenic *Arabidopsis* plants overexpressing *TaPP2AbB"-α* have a more developed root system than control plants under normal conditions. Previous studies showed that root growth can be affected by environmental conditions and genetic characters [42], such as moderate soil drying which promotes the elongation of lateral roots [43]. Overexpression of *TaSnRK2.3–GFP* in *Arabidopsis* also generated an improved root system, expressed by longer primary root and more lateral roots [12]. In this study, *TaPP2AbB"-α*-overexpressing *Arabidopsis* lines developed more lateral roots and stronger primary roots than the control under normal conditions, suggesting that *TaPP2AbB"-α* is involved in regulation of root growth and development.

Under medium stresses, i.e. 100 mM NaCl and 200 mM mannitol, both transgenic lines developed more lateral roots (Figure 5), implying that the plants can spontaneously adapt to certain environmental stress, and that this phenotype may also represent a common adaptive response to diverse stresses among plants. As in rice, water deficit also results in increased lateral root formation [44]. In addition, more lateral roots were produced under salt stress in wild-type *Arabidopsis* [45]. Previous studies showed that *Arabidopsis* developed more lateral roots under 100 mM NaCl, but less lateral roots under 125 mM NaCl [17]. A more developed root system helps plants to improve the efficiency of water and nutrient uptake, especially under abiotic stress [46]. With more lateral roots and longer primary roots, more water and nutrient uptake may be available in transgenic *Arabidopsis*. This could be the reason why overexpression lines of TaPP2AbB"-α grew better than control plants, even under NaCl and mannitol stresses.

The transcript expression level of *TaPP2AbB"-α* is stronger in roots than in leaves of wheat seedlings, suggesting that *TaPP2AbB"-α* mainly functions in the root system. This inference also matches the phenotypes of overexpression lines of *TaPP2AbB"-α* in *Arabidopsis*. The expression of *TaPP2AbB"-α* is up-regulated by multiple stresses (Figure 6). Previous studies showed that PP2A, or specific PP2A subunits, function as transducers of osmotic stress and in low temperature signaling [47]. In *Saccharomyces cerevisiae*, PP2AbB′ is an essential activating factor for stress gene transcription, such as *CTT1* (encoding cytosolic catalase T), *HSP12* (encoding a small heat shock protein), and *PGM2* (encoding phosphoglucomutase 2) [48]. Moreover, expression of genes encoding PP2Ac subfamily I is induced by drought and NaCl stress [49], [50]. *TaPP2Ac1*, which clades in PP2Ac subfamily II, is induced by NaCl, PEG, low temperature and ABA [51]. Considering these findings, the role of *PP2Ac* in stress signaling matches with the expression of *TaPP2AbB"-α* under multiple environmental stresses. Together with the phenotypic data for *TaPP2AbB"-α* overexpression lines, we surmise that transgenic plants develop more lateral roots when the transcript level of *TaPP2AbB"-α* is induced by osmotic stress.

PP2A is highly conserved and the core enzyme interacts with a variable regulatory subunit to form a PP2A holoenzyme. Human PP2Aa interacts with PP2Ac to form a stable AC core dimer through its intra-repeat loops and inner helices, and B56 (PP2AbB′) interacts with both PP2Aa and PP2Ac [52]. Here, TaPP2AbB"-α exhibits the same subcellular localization pattern with TaPP2Aa (unpublished data) and TaPP2Ac [51]. As PP2A is a heterotrimeric protein, we detected interactions of TaPP2AbB"-α with PP2Aa and PP2Ac. As shown in Figure 7, the interacting relationships of TaPP2AbB"-α with TaPP2Aa and TaPP2Ac were verified in both yeast and tobacco leaves. Moreover, we found that TaPP2Aa has high homology with RCN 1 in *Arabidopsis* (Figure S1). The mutant line of *rcn1* (pp2aa1) is sensitive to osmotic stress and developed less lateral root than the normal plants [53]. RCN1 modulates polar auxin transport (PAT), and dephosphorylates auxin efflux carrier PIN proteins to influence the distribution of auxin accompanied with Ser/Thr kinase PINOID [15], [16]. TaPP2Ac is in the same clade with PP2Ac subfamily II (PP2Ac3 and PP2Ac4) in *Arabidopsis* (Figure S2), and a double mutant of PP2Ac, *pp2ac3*/*pp2ac4* exhibits defective root development in *Arabidopsis*. PP2Ac subfamily II is also involved in the auxin signaling pathway [20]. Interaction and the phenotype of *PP2Aa* and *PP2Ac* suggest that positive regulation of *TaPP2AbB"-α* in root development may be associated with auxin signaling.

In this study, TaPP2AbB"-α enhanced root development in overexpressing *Arabidopsis* under normal conditions. With onset of environmental stress, TaPP2AbB"-α is induced to promote increased root growth, and thus enhanced absorbsion efficiency in the uptake of water and nutrients for survival. In this process, PP2Aa and PP2Ac may interact with PP2AbB"-α and then function as a holoenzyme to act on certain substrates. More evidence needs to be obtained to uncover the molecular mechanism by which *TaPP2AbB"-α* enhances osmotic-stress tolerance.

## Supporting Information

Figure S1 **Alignment of the amino acid sequences of PP2AbB"-α from wheat and** ***Arabidopsis*** **.** Alignment was performed according to DNAman. The accession number of AtPP2Aa1 is NP_173920.1, the accession number of TaPP2Aa is AEB40165.1. Common identical amino acid residues are shown in black background. The HEAT motif is marked in red rectangles. Abbreviations on the left side of the sequence are: At, *Arabidopsis thaliana*; Ta, *Triticum aestivum*.
(TIF)

Figure S2 **Phylogenetic tree of TaPP2Ac from wheat and PP2Ac from** ***Arabidopsis*** **.** This phylogenetic tree is performed by MEGA 5.05. TaPP2Ac, accession number: ABO16371.1; AtPP2Ac-1, NP 176192.1; AtPP2Ac-2; NP 172514.1; AtPP2Ac-3, NP 567066.1; AtPP2Ac-4, NP 565974.1; AtPP2Ac-5, NP 172514.1. Bootstrap values are in percentages. Abbreviations on the right side of the tree are: At, *Arabidopsis thaliana*; Ta, *Triticum aestivum*.
(TIF)
