# Supplementary figures and images for: Cloning and Characterization of TaPP2AbB"-α, a Member of the PP2A Regulatory Subunit in Wheat
Source: PLoS One. 2014 Apr 7;9(4):e94430. doi: 10.1371/journal.pone.0094430 (PMC3978047; doi:10.1371/journal.pone.0094430)

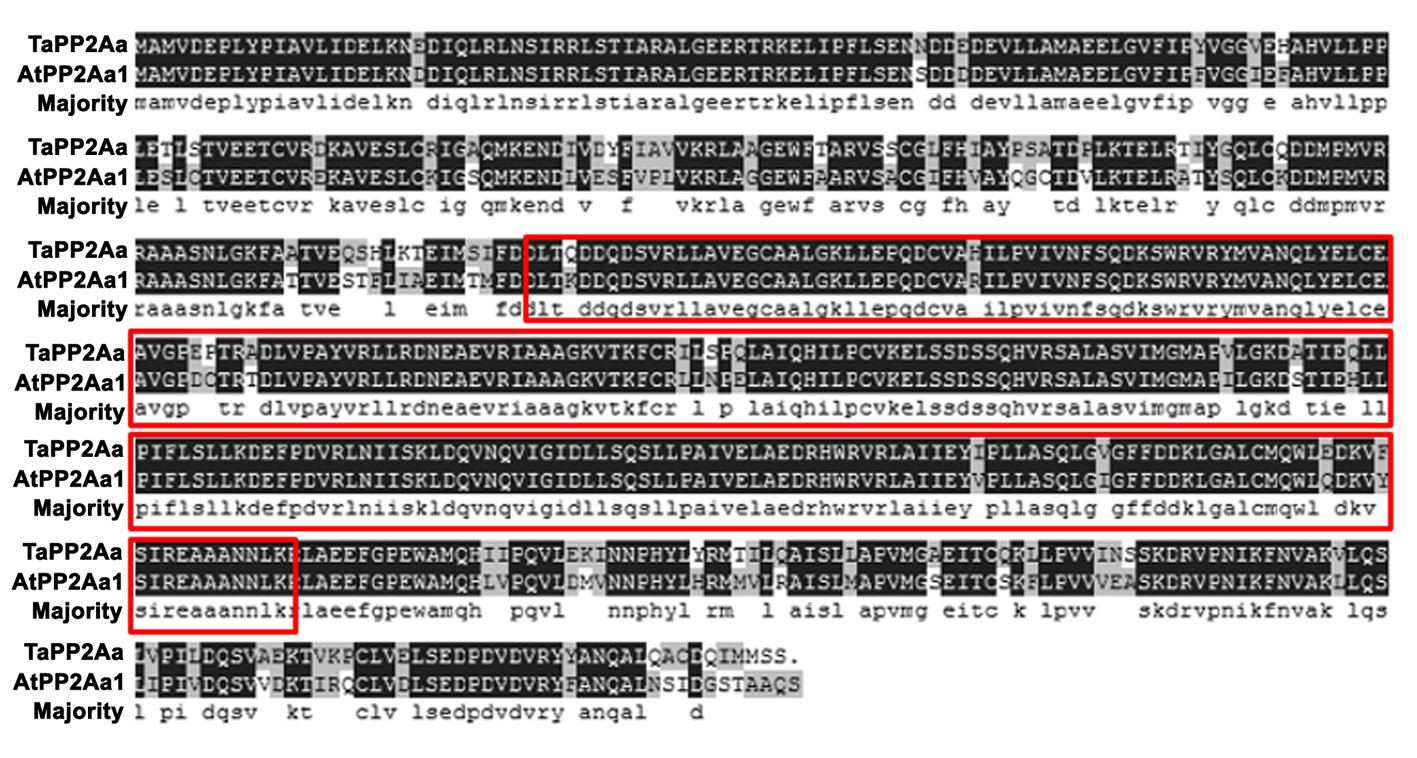

Supplement: Figure S1 — Alignment of the amino acid sequences of PP2AbB"-α from wheat and Arabidopsis . Alignment was performed according to DNAman. The accession number of AtPP2Aa1 is NP_173920.1, the accession number of TaPP2Aa is AEB40165.1. Common identical amino acid residues are shown in black background. The HEAT motif is marked in red rectangles. Abbreviations on the left side of the sequence are: At, Arabidopsis thaliana; Ta, Triticum aestivum. (TIF) [file pone.0094430.s001.tif]

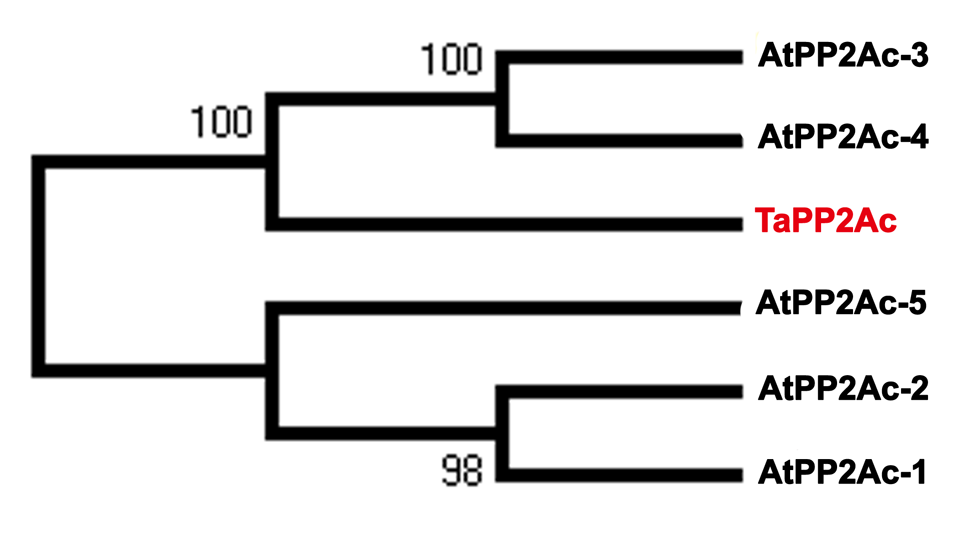

Supplement: Figure S2 — Phylogenetic tree of TaPP2Ac from wheat and PP2Ac from Arabidopsis . This phylogenetic tree is performed by MEGA 5.05. TaPP2Ac, accession number: ABO16371.1; AtPP2Ac-1, NP 176192.1; AtPP2Ac-2; NP 172514.1; AtPP2Ac-3, NP 567066.1; AtPP2Ac-4, NP 565974.1; AtPP2Ac-5, NP 172514.1. Bootstrap values are in percentages. Abbreviations on the right side of the tree are: At, Arabidopsis thaliana; Ta, Triticum aestivum. (TIF) [file pone.0094430.s002.tif]
